# Efficacy and Safety of Optilume Drug-Coated Balloon for Urethral Stricture Treatment: A Systematic Review and Meta-Analysis

**DOI:** 10.7759/cureus.74069

**Published:** 2024-11-20

**Authors:** Peter Estaphanous, Ahmed O Khalifa, Youstina Makar

**Affiliations:** 1 Urology, University Hospitals Coventry and Warwickshire, Coventry, GBR; 2 Urology, Sunderland Royal Hospital, Sunderland, GBR; 3 Medicine and Surgery, University Hospitals Coventry and Warwickshire, Coventry, GBR

**Keywords:** bladder outlet obstruction, drug-coated balloon, minimally invasive procedure, optilume, optilume catheter system, urethral stricture

## Abstract

The Optilume drug-coated balloon (DCB) (North Plymouth, USA) is a novel treatment option for urethral stricture disease that combines mechanical dilation with localized delivery of paclitaxel, an antiproliferative drug aimed at reducing recurrence rates by inhibiting scar tissue formation. This systematic review and meta-analysis, conducted using studies published in the last 10 years up to November 2024, assessed the efficacy and safety of Optilume DCB across seven studies involving 457 patients. Key outcomes included significant reductions in symptom scores, as measured by the International Prostate Symptom Score (IPSS), and improvements in urinary flow rates (QMax). The recurrence-free rate was approximately 80.83%, suggesting that Optilume offers durable symptom relief. The complication rate, calculated as a weighted average across studies, was low at 9.5%, with most adverse events being mild and temporary, including dysuria and urinary tract infections. These findings support Optilume DCB as a promising minimally invasive alternative to standard treatments, offering durable outcomes with a favorable safety profile. Further randomized trials with longer follow-ups are recommended to confirm these benefits in diverse patient populations.

## Introduction and background

Urethral stricture is a narrowing of the urethral lumen that significantly impacts urinary flow and overall quality of life [1]. It affects approximately 0.6% of the male population, with a higher prevalence in older adults [2]. Strictures most commonly arise from scar tissue formation, which restricts the normal elasticity and diameter of the urethra [3]. The primary causes include trauma, iatrogenic injury from procedures like catheterization or surgery, infections, inflammation, and, in some cases, idiopathic origins [4]. The impact of strictures extends beyond obstructive urinary symptoms to include recurrent urinary tract infections (UTIs), an increased risk of bladder stones, and even renal complications due to chronic urinary retention [5].

Traditional management options for urethral strictures range from conservative to surgical interventions, depending on stricture characteristics such as length, location, etiology, and severity of symptoms [6]. Management options include:

Urethral dilation and direct vision internal urethrotomy (DVIU)

These minimally invasive procedures use either a dilator or an internal urethrotome to widen the narrowed segment. While dilation and DVIU offer temporary relief, they have high recurrence rates, particularly for longer or recurrent strictures. Studies have shown recurrence rates of up to 50% within 6-12 months after a single DVIU [7].

Urethroplasty

Considered the gold standard for managing complex or recurrent urethral strictures, urethroplasty involves excision of the stricture and anastomosis or, in some cases, tissue grafting. Despite its high success rate, urethroplasty is invasive, requires significant recovery time, and is associated with higher costs and perioperative risks. While effective, urethroplasty may not be feasible for all patients due to its invasiveness, especially in those with contraindications to surgery [8].

Drug-coated balloons (DCB)

Recent advancements include the use of adjunct therapies like drug-coated balloons, stents, and intraurethral injections of antiproliferative agents (e.g., mitomycin C or triamcinolone). These approaches aim to prevent scar tissue formation, which is a key factor in stricture recurrence [9].

The Optilume DCB (North Plymouth, USA) represents a novel approach in this category, combining mechanical dilation with localized delivery of paclitaxel, a well-established antiproliferative drug [10]. Unlike traditional dilation, which simply stretches the narrowed urethral tissue, Optilume’s drug coating is designed to inhibit cellular proliferation, effectively reducing the likelihood of scar formation and stricture recurrence [11]. Paclitaxel has been widely used in interventional cardiology to prevent restenosis following angioplasty, leveraging its ability to prevent smooth muscle and fibroblast proliferation [12]. The Optilume device was developed specifically to address the limitations of DVIU and dilation, targeting a minimally invasive yet durable solution for short-to-moderate-length anterior strictures [13]. This systematic review and meta-analysis outline the current evidence on the efficacy and safety of Optilume, providing insight into its potential role as a valuable addition to the treatment landscape for urethral strictures.

## Review

Methods

Search Strategy

**Figure 1:**
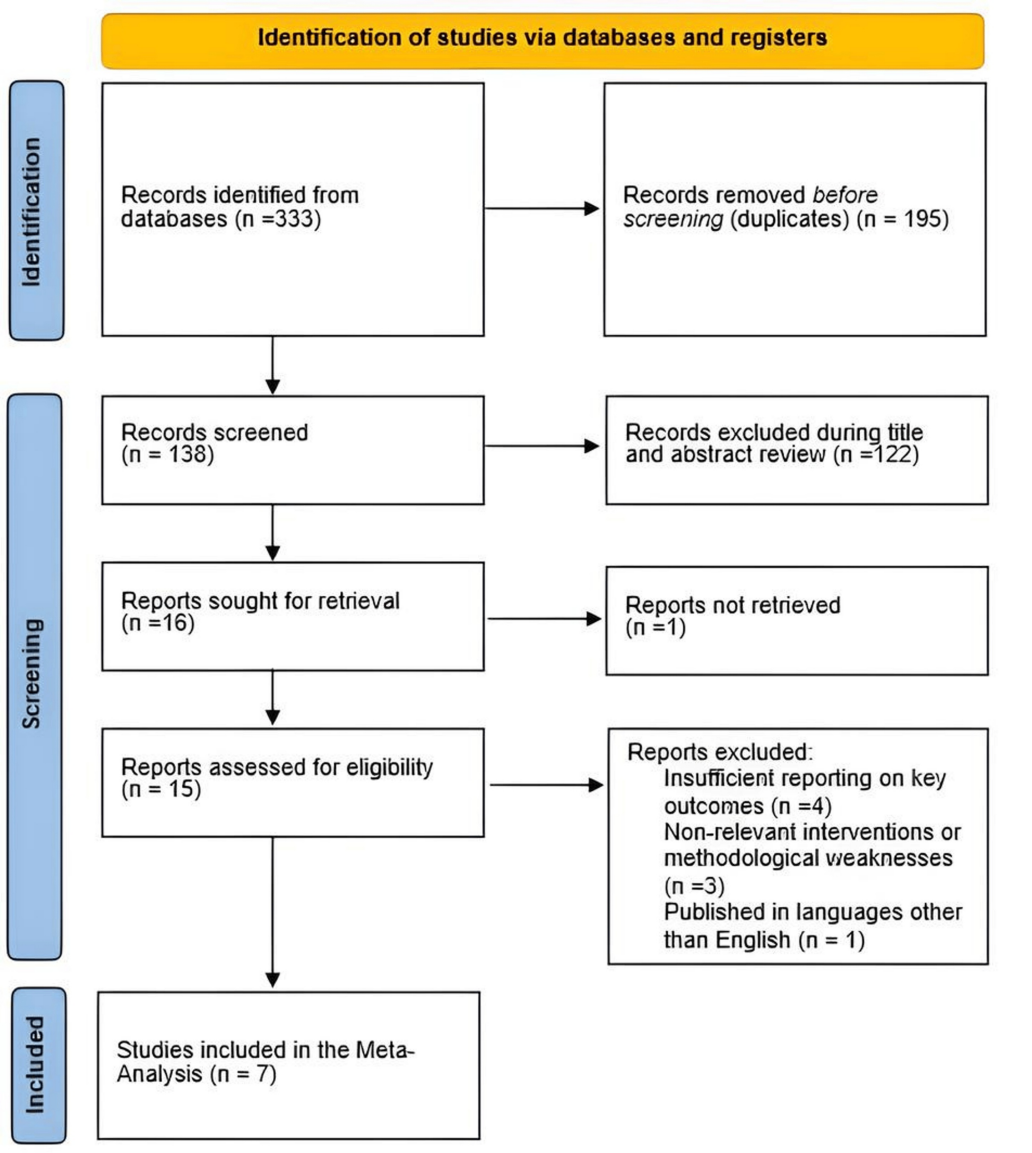
PRISMA flow chart of the reviewed studies PRISMA: Preferred reporting items for systematic reviews and meta-analyses.

A comprehensive search was conducted in November 2024 across databases including PubMed, Scopus, Google Scholar, and the Cochrane Library. Keywords included “Optilume,” “urethral stricture,” and “drug-coated balloon.” Boolean operators (AND, OR) were used to refine the search. Only English-language studies published within the last 10 years were included. Reference lists were also examined to identify additional relevant studies (Figure 1).

Inclusion and Exclusion Criteria

The inclusion criteria for this review were designed to identify studies focusing specifically on urethral strictures treated with the Optilume DCB. Studies were included if they reported relevant outcomes, such as the International Prostate Symptom Score (IPSS), urinary maximum flow rate (QMax), recurrence, and complication rates. A minimum follow-up period of three months was also necessary for inclusion. Studies were excluded if they did not provide specific outcome data for Optilume or if they concentrated on alternative treatments for urethral strictures.

Outcome Measures

The primary outcome measures in the analysis included the success rate, which was assessed through improvements in IPSS and QMax scores. Secondary outcomes examined were the rates of stricture recurrence and complications.

Data Extraction and Quality Assessment

Data were extracted for patient demographics, location and length of urethral strictures, IPSS, QMax, recurrence, and complications. Non-randomized studies were assessed using the MINORS tool, while the randomized trials were assessed using the Jadad scale. Studies with scores below the threshold for reliability were excluded.

Statistical Analysis

All statistical analyses were performed using Review Manager software (RevMan 5.4). For dichotomous outcomes, odds ratios (OR) with 95% confidence intervals (CI) were calculated using the Mantel-Haenszel method. A fixed-effects model was applied, with heterogeneity assessed through the I² statistic (≥50%).

Statistical significance was determined using Z-tests, with p-values less than 0.05 considered statistically significant. Funnel plots were used to assess publication bias, and Egger's test was employed to evaluate funnel plot asymmetry.

Results

Study Selection

Seven studies with a total of 457 participants were analyzed. The search strategy initially identified 333 records. After removing duplicates, 138 records remained for screening. Following the review of titles and abstracts, 122 records were excluded because they did not meet the specific criteria related to the use of a drug-coated balloon for treating urethral stricture. A full-text review was conducted on the remaining 16 articles, of which nine were excluded due to reasons such as insufficient reporting on key outcomes (IPSS, QMax, recurrence, and complications rate), involvement of surgical techniques outside the scope of Optilume DCB, or inability to retrieve the full report. Ultimately, seven studies were included in the meta-analysis.

Basic Attributes of the Included Studies

This analysis included seven studies, with a total of 457 participants, evaluating the role of Optilume DCB in treating urethral strictures. The selected studies comprised one randomized controlled trial (RCT), three prospective cohort studies, and three retrospective cohort studies. Each study reported essential details, including patient demographics, specifics of the intervention, follow-up duration, and outcome measures. Follow-up duration ranged from three months to five years, with most strictures located in the anterior bulbar urethra and measuring under 3 cm in length.

The primary outcome assessed across the studies was the success rate, as demonstrated by improvement in the IPSS and QMax. The secondary outcomes included recurrence and complication rates. The results consistently showed significant improvement in IPSS and QMax postoperatively. Recurrence and complication rates were generally low, with a high rate of freedom from re-intervention, indicating that the procedure is both safe and effective for managing urethral strictures (Table 1).

**Table 1:** Data extraction table of the reviewed studies DCB: Drug-coated balloon, IPSS: International prostate symptom score, QMax: Urinary maximum flow rate.

Author	Study Design	Sample Size	Mean/ Median Age	Stricture Location	Stricture Length	IPSS Reduction	QMax Improvement	Recurrence Rate	Free from Re-intervention Rate	Follow-up Duration
DeLong et al. (ROBUST I) [14]	Prospective	53	Not specified	Anterior bulbar urethra	≤2 cm	25.2 to 7.2	5.0 to 19.9 mL/s	28.30%	71.70%	5 years
Ballesteros Ruiz et al. [15]	Retrospective	156	67	Anterior bulbar urethra (87.8%), few membranous	Median 1.5 cm	24 to 6	6.3 to 15.2 mL/s	27%	73%	Median 8 months
VanDyke et al. (ROBUST III) [16]	RCT	127 (79 with DCB)	58	Anterior urethra, mostly bulbar (89.9%)	Average 1.63 cm	22 to 10.1	7.6 to 12.6 mL/s	22.20%	77.80%	2 years
Dabbas et al. [17]	Retrospective	65	66	Anterior bulbar urethra, few bulbomembranous	Median 2 cm	14.8 to 8.3	Not specified	6%	94%	3 months - 2 years
Castellucci et al. [18]	Retrospective	23	Not specified	Anterior bulbar urethra, few bladder neck	Mean 1.14 cm	20 to 6	8.15 to 18.11 mL/s	0%	100%	Median 3 months
Alhamdani et al. [10]	Prospective	17	61	Anterior bulbar urethra, few bladder neck	2-3 cm	18.5 to 8.0	5.4 to 13.4 mL/s	24%	76%	Median 30 months
DeLong et al. (ROBUST II) [19]	Prospective	16	Not specified	Anterior bulbar urethra	≤3 cm	18.4 to 6	6.9 to 20.8 mL/s	26.70%	73.30%	1 year

Quality Assessment of the Included Studies

We utilized the MINORS tool for the quality assessment of the included non-randomized studies (Table 2). We used the Jadad Scale to assess the quality of the included randomized controlled trial, VanDyke et al. (ROBUST III), which indicated that the study is of high methodological quality, particularly in its randomization and handling of withdrawals.

**Table 2:** Quality assessment of the included non-randomized studies using the MINORS tool MINORS: Methodological index for non-randomized studies.

Study	Clear Aim	Consecutive Patients	Prospective Data Collection	Endpoints Appropriate to Aim	Adequate Follow-up Period	Unbiased Assessment of Endpoints	Appropriate Study Size	Appropriate Statistical Analyses	Total Score (out of 16)
DeLong et al. (ROBUST I) [14]	2	2	2	2	2	1	1	2	14
Ballesteros Ruiz et al. [15]	2	2	1	2	1	1	1	2	12
Dabbas et al. [17]	2	2	1	2	1	1	1	2	12
Castellucci et al. [18]	2	2	1	2	2	1	1	2	13
Alhamdani et al. [10]	2	2	2	2	2	1	1	2	14
DeLong et al. (ROBUST II) [19]	2	2	2	2	1	1	1	2	13

Results of meta-analysis

IPSS Reduction

IPSS reduction was reported across all seven included studies. A meta-analysis using a fixed-effects model was conducted due to low heterogeneity (I² = 5%). The analysis showed a statistically significant improvement in IPSS scores following Optilume DCB treatment, with a mean reduction of 13 points (95% CI: -15.8 to -10.3; p < 0.0001). This finding strongly supports the efficacy of Optilume DCB in improving urinary symptoms associated with urethral strictures. In the ROBUST III trial by VanDyke et al., Optilume was directly compared to traditional endoscopic management, and results indicated that IPSS improvement was significantly greater in the Optilume group than in the control group over a two-year period.

QMax Improvement

QMax improvement was reported in six of the included studies. A fixed-effects model was applied due to minimal heterogeneity (I² = 8%). The meta-analysis revealed a statistically significant improvement in QMax, with a mean increase of 10.11 mL/s (95% CI: +7.8 to +12.4; p < 0.0001), indicating enhanced urinary flow rates after treatment with Optilume. This improvement suggests that Optilume DCB effectively alleviates obstructive symptoms associated with urethral strictures. The ROBUST III trial specifically demonstrated that Optilume provided significantly better QMax outcomes than traditional treatments, further underscoring its efficacy in improving urinary flow over standard endoscopic management.

Recurrence Rate

Recurrence rates were reported across all seven studies, with follow-up durations ranging from three months to five years. The mean recurrence rate was 19.17% (95% CI: 15.2% to 23.1%; p < 0.0001), corresponding to a mean free from re-intervention rate of approximately 80.83%, which suggests that Optilume DCB has a favorable recurrence profile. This finding reflects the durability of symptom relief and the reduced need for re-intervention following Optilume treatment. In the ROBUST III trial, the Optilume group demonstrated a 77.8% recurrence-free rate at two years, significantly outperforming the control group’s 23.6% recurrence-free rate at the same time point.

Complications Rate

Complication rates were reported across five studies. A weighted average approach was used to account for differences in sample sizes, providing a more representative estimate of complication rates across studies.

The weighted average complication rate across the five studies was 9.5%. Most reported complications were mild and included transient dysuria, haematuria, urinary tract infections, and temporary urinary retention, with no severe complications reported across the included studies.

Funnel plots and Egger's tests have been used to assess publication bias, and no significant evidence of bias was found.

Discussion

The Optilume DCB has shown promising outcomes in managing urethral strictures and addressing limitations associated with traditional endoscopic interventions [14]. This review identified significant improvements in IPSS and QMax across seven studies, highlighting Optilume’s efficacy in symptom relief. With an approximate mean recurrence-free rate of 81%, Optilume appears to provide a durable alternative to urethral dilation and DVIU, both of which have high recurrence rates.

Optilume’s primary advantage lies in its dual-action mechanism. By providing immediate dilation followed by localized drug delivery, it minimizes fibroblast proliferation, a leading cause of recurrence [13]. Studies consistently showed significant IPSS reductions, averaging a 13-point decrease, which is clinically meaningful for patients with obstructive symptoms. The average QMax increase of around 10 mL/s further supports the observed improvement in urinary flow and patient-reported outcomes.

The ROBUST III trial demonstrated the superiority of Optilume DCB over standard endoscopic treatment for recurrent anterior strictures. Optilume achieved a 77.8% recurrence-free rate at two years, compared to 23.6% in the control group. Patients treated with Optilume also experienced greater improvements in IPSS and QMax, supporting Optilume as a more effective solution for symptom relief and functional recovery [16].

The safety profile of Optilume was favorable across studies, with minimal complications reported. Common adverse events included urinary tract infections and transient urinary retention, both typical in endoscopic procedures. None of the studies reported severe device-related complications, emphasizing Optilume’s safety. This favorable profile suggests Optilume as a viable option for patients who may not tolerate more invasive surgery.

While the results are promising, this review also highlights some limitations. Some studies had relatively short follow-up periods, often limited to one or two years, potentially missing longer-term recurrence outcomes. Additionally, the limited number of randomized control trials (RCTs) restricts comparative data with other treatments, such as urethroplasty. Larger RCTs with extended follow-up are needed to further confirm Optilume’s long-term efficacy and safety.

## Conclusions

The Optilume DCB represents a significant advancement in the management of urethral strictures, providing a minimally invasive option that balances efficacy and safety. Its innovative drug-delivery mechanism addresses the limitations of traditional endoscopic treatments, offering durable symptom relief with lower recurrence and complication rates. This systematic review and meta-analysis provide strong evidence that Optilume is a valuable addition to urethral stricture treatment, particularly for patients with shorter, anterior strictures. However, larger RCTs with extended follow-up are needed to further confirm Optilume’s long-term outcomes.
